# Interactions of warfarin with COVID-19 vaccine/drugs, monoclonal antibodies, and targeted anticancer agents from real-world data mining

**DOI:** 10.3389/fphar.2024.1418997

**Published:** 2024-11-29

**Authors:** Yan Gao, Qingsong Huang, Jun Li, Junsheng He, Fang Qian, Juanjuan Yi

**Affiliations:** ^1^ Department of Pharmacy, Shanghai Jiading District Hospital of Traditional Chinese Medicine, Shanghai, China; ^2^ Department of Internal Medicine, Shanghai Jiading District Hospital of Traditional Chinese Medicine, Shanghai, China; ^3^ Department of Infectious Diseases and Public Health, City University of Hong Kong, Hong Kong SAR, China; ^4^ School of Data Science, City University of Hong Kong, Hong Kong SAR, China

**Keywords:** drug-related problem (DRP), adverse drug reaction (ADR), warfarin, vitamin K antagonist (VKA), drug-drug interaction (DDI), bleeding, safety, risk

## Abstract

**Objective:**

This study aims to examine the unresolved drug-drug interactions of warfarin using real-world data.

**Methods:**

Electronic medical records from a hospital in Shanghai, China, were used to summarize drug-related problems (DRPs) among inpatients taking warfarin in 2022. Additionally, adverse event data for warfarin from January 2004 to December 2023 were extracted from the U.S. adverse event reporting system to evaluate the bleeding risk associated with the concomitant use of warfarin and COVID-19 Vaccine/drugs, monoclonal antibodies, and targeted anticancer agents.

**Results:**

The electronic clinical records yielded 180 cases, of which 130 cases (72.22%) had 276 DRPs identified. DRP5 (n = 172, 62.32%) was identified as the most common issue, comprising 145 drug interactions and 27 adverse drug reactions (ADRs). The analyses of warfarin ADR records (n = 53,709) from the database revealed that tocilizumab (adjusted Odds Ratio (aOR): 3.39 [95% CI: 1.77–7.03]; *P* < 0.001), ibrutinib (aOR: 2.53 [1.61–4.19]; *P* < 0.001), and cabozantinib (aOR: 3.34 [1.40–9.85]; *P* = 0.013) significantly increased the risk of warfarin bleeding. In contrast, nirmatrelvir–ritonavir (aOR: 0.32 [0.14–0.69]; *P* = 0.004), adalimumab (aOR: 0.72 [0.56–0.93]; *P* = 0.012), golimumab (aOR: 0.18 [0.05–0.50]; *P* = 0.002), tofacitinib (aOR: 0.51 [0.29–0.86]; *P* = 0.013), and dabrafenib (aOR: 0.17 [0.04–0.55]; *P* = 0.007) significantly reduced the risk of bleeding when combined with warfarin. Remdesivir combined with warfarin was associated with a statistically significant increase in bleeding events (*P* = 0.047); while the risk was not significant after adjusting for age and sex (aOR: 1.79; *P* = 0.2). No significant effect was observed with the COVID-19 vaccine (aOR: 0.89; *P* = 0.8).

**Conclusion:**

Drug-drug interactions contribute to the adverse effects of warfarin. This study provides real-world evidence of newly identified drug interactions with warfarin. It reminds clinicians to monitor INR and adjust warfarin doses accordingly when used in combination with these medications.

## 1 Introduction

Warfarin is a classic oral anticoagulant in clinical use for nearly 70 years (Patriquin and Crowther, 2011; Muse et al., 2022). It effectively mitigates the risk of thromboembolism in patients with atrial fibrillation, deep vein thrombosis, and other thrombotic conditions (Hindricks et al., 2021; Tan and Lee, 2021). Warfarin comprises of R and S enantiomers, with the efficacy of S-warfarin being approximately 2–5 times that of R-warfarin (Fasco and Principe, 1982). S-warfarin is almost predominantly metabolized by the CYP2C9 enzyme in the liver. Warfarin exhibits significant individual variability, a narrow therapeutic window, International Normalized Ratio (INR) monitoring requirements, and frequent drug-drug interactions (DDIs). However, DDIs in warfarin cannot be determined from randomized trials, and always gathered from the real-world clinical experience. Various medications, including NSAIDs/COX-2 inhibitors, Antidepressants, Antibacterials/antiprotozoals, Acetylsalicylic acid, Fluvastatin, Simvastatin, Allopurinol, Paracetamol, Corticosteroids, Omeprazole, Amiodarone, and herbs, have been reported to potentiate or inhibit the effects of warfarin (Narum et al., 2011; Tan and Lee, 2021). Ongoing research aims to identify new drug interactions to prevent side effects. The interactions between warfarin and some recently marketed drugs, such as COVID-19 vaccine/drugs, monoclonal antibodies, and targeted anticancer drugs, have yet to be clearly defined.

The term drug-related problems (DRPs) refers to events or conditions related to drug treatment that may interfere with desired health outcomes, such as inappropriate drug selection, adverse drug events, and DDIs (da Costa et al., 2016; Pfister et al., 2017). The Second Consensus of Granada is internationally recognized as a valuable tool for categorizing DRPs into necessity, efficacy, and safety (da Costa et al., 2016). This systematic approach helps to identify potential issues in the drug treatment process (Martinez-Aguilar et al., 2023). The FDA Adverse Event Reporting System (FAERS) serves as a global database that collects reports of adverse drug events and provides data to support postmarketing drug safety. The database contains information on patient demographics, drugs, indications, outcomes, and reactions. It is extensively utilized for postmarketing studies of adverse drug reactions (ADR) and DDIs (Yao et al., 2020; Goldman et al., 2021). COVID-19 therapeutics represent a new class of drugs, most of which were approved under Emergency Use Authorization to address the urgent needs of the pandemic. Owing to the accelerated approval process and short duration of clinical use for these drugs, long-term safety data is lacking, leaving the full scope of potential adverse effects and DDIs unclear.

To summarize the epidemiological characteristics of warfarin and assess its potential interactions in a clinical setting, we retrospectively analyzed DRPs in 180 Electronic Medical Record (EMR) of patients with warfarin from January to December 2022. We also used FAERS data to evaluate interactions that affect warfarin anticoagulation. This study provides real-world evidence for the clinical safety of warfarin.

## 2 Materials and methods

### 2.1 Data source

The data for this retrospective analysis was collected from the EMRs of Shanghai Jiading District Hospital of Traditional Chinese Medicine. Overall, 180 inpatients who were administered warfarin (Shanghai Xinyi Pharmaceutical Co., Ltd.) during their hospitalization from January to December 2022 were included in the study. The adverse reaction data for warfarin were collected from the U.S. AERS and FAERS datasets, covering 20 years of adverse events from Q1 2004 to Q4 2023.

### 2.2 Data processing

**FIGURE 1:**
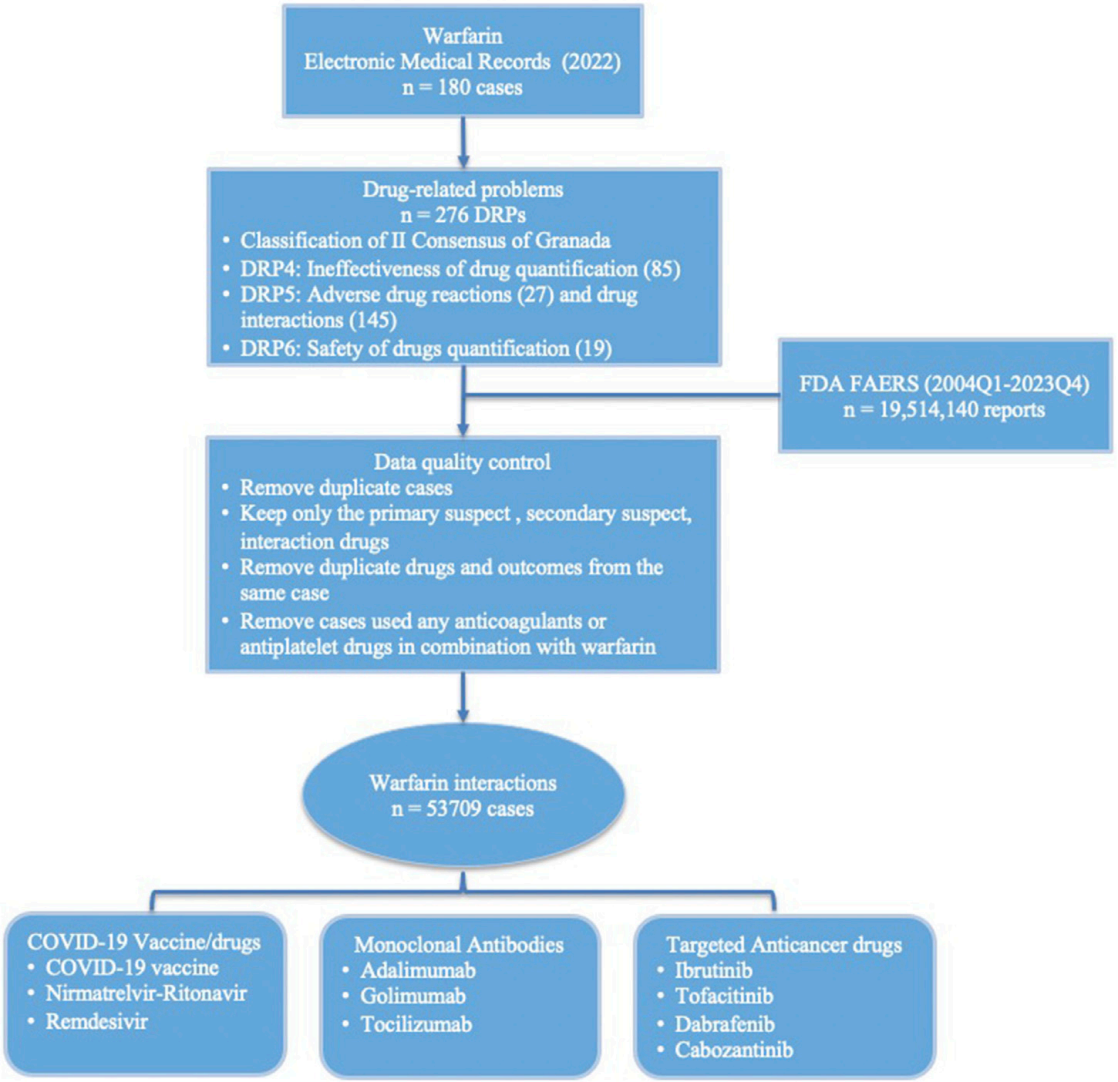
Data processing and analysis workflow.

To obtain high quality data, all adverse event reports that identified warfarin as the primary suspect, secondary suspect, or interaction were selected (Figure 1). Multiple repeated reports of the same adverse event were excluded, retaining only the most recent report; multiple outcomes of the same adverse event were removed, retaining only the most severe outcome. Furthermore, cases that used any anticoagulants or antiplatelet drugs in combination with warfarin, such as heparin, dabigatran, rivaroxaban, apixaban, edoxaban, aspirin, prasugrel, ticagrelor, vorapaxar, and clopidogrel were removed from the dataset. The suspected interaction drugs were selected from the reports labeled as interaction events, and the drug names that included only warfarin and one other drug were extracted. The names of these drugs were manually corrected, while the terms of adverse events were standardized and categorized with the MedDRA (v26.0).

### 2.3 Statistical analysis

The EMR data collected to summarize DRPs were classified using the Second Consensus of Granada (Table 1). The clinical characteristics of patients who were administered warfarin were assessed in this study, including age, sex, daily dose, duration of treatment, department, ADRs, indication, and DRPs.

**TABLE 1:** Criteria for the second consensus of granada.

Classification		Drug related problems	Performance
Necessity	DRP1	The disease has not been treated and the required drug treatment has not been obtained	The required drugs were not used
Effectiveness	DRP2	Use unnecessary drugs, medication is not correct	There is no indication for drug use, and the inappropriate dosage form is selected
	DRP3	Non-quantitative ineffectiveness of drugs	Use wrong drugs and choose inappropriate solvents
	DRP4	Ineffectiveness of drug quantification	The drug dose is too low, the frequency of use is too low, and the course of treatment is inadequate
Security	DRP5	Non-quantitative safety of drugs	Patients have their own specific diseases and use drugs with potential safety problems on their own, incompatibility, contraindications, adverse drug reactions, too little solvent, inappropriate drug interaction and route of administration
	DRP6	Safety of drugs quantification	Repeated use of drugs, drug dosage is higher than the specified daily dose, the course of treatment duration is too long, and the frequency is higher than the normal value

Haemorrhage, which is the most common adverse reaction associated with warfarin pharmacology, is a manifestation of its overdose or potentiation. We investigated the interactions of warfarin with 10 drugs by selecting bleeding events as the outcome. Bleeding events were categorized according to the MedDRA, including the following: “bruising, ecchymosis and purpura,” “gastrointestinal haemorrhagesnec,” “menstrual cycle and uterine bleeding disorders,” “haemorrhagesnec,” “nervous system haemorrhagic disorders,” ocular haemorrhagic disorders,” “renal haemorrhagic disorders,” “reproductive system haemorrhages,” “gastrointestinal haemorrhages,” “gastric and oesophagealhaemorrhages” from High Level Terms (HLT), and “haemorrhage terms (excl laboratory terms)” and “haemorrhage laboratory terms” from Standardized MedDRA Queries (SMQ). The difference between categorical variables was analyzed using the Chi-square test. Logistic regression analysis was used to determine whether co-administered drugs influenced bleeding. Multivariate analysis was adjusted for sex (female and male) and age (<65 and ≥65). Data cleaning was performed using Python 3.6.0, and statistical analyses were conducted using R 4.2.0. A statistically significant result was defined as a two-tailed *P*-value of less than 0.05.

## 3 Results

### 3.1 Clinical characteristics and drug-related problems

Of the 180 inpatients receiving warfarin from the EMRs, 91 were female, accounting for 50.56% (Table 2). The mean age of the patients was 75.24 ± 11.69 years, ranging from 41 to 96 years. Most patients were elderly, aged ≥65 years, accounting for 56.67%. The most common clinical indication for warfarin was non-valvular atrial fibrillation, identified in 87 patients (48.33%).

**TABLE 2:** Clinical characteristics of inpatients using warfarin from electronic medical record (2022).

Characteristics	Number, n	Proportion, %
Number of cases	180	100.0
Sex		
Male	89	49.4
Female	91	50.6
Age (years)		
0–18	0	0.0
19–64	78	43.3
≥65	102	56.7
Dose daily (mg)		
≤2.5	167	92.8
>2.5 and ≤5	13	7.2
Duration (days)		
≤7	21	11.7
>7 and ≤30	159	88.3
Clinical indication		
Non valvular atrial fibrillation	87	48.3
Pulmonary embolism	35	19.4
Deep venous embolism	32	17.8
Arterial embolism	26	14.5
Department		
Cardiology department	66	36.7
Respiratory department	40	22.2
Oncology department	33	18.3
Endocrinology and Gastroenterology department	27	15.0
Neurology department	14	7.8
Drug related problems (DRPs)	276	100.0
DRP1	0	0.0
DRP2	0	0.0
DRP3	0	0.0
DRP4	85	30.8
DRP5	172	62.3
DRP6	19	6.9
Adverse reaction (ADR)	27	100.0
Blood urine	5	18.5
Gastrointestinal haemorrhage	15	55.6
Ecchymosis	4	14.8
Haemoptysis	3	11.1
Combined drugs in ADR cases	27	100.0
0–3	0	0.0
>3 and ≤5	7	25.9
>5	20	74.1

EMR, electronic medical record.

In this study, 130 of 180 patients (72.22%) experienced 276 DRPs (Table 2). Among these, DRP4, DRP5, and DRP6 occurred 85 (30.80%), 172 (62.32%), and 19 (6.88%) times, respectively (refer to Table 2). DRP4 resulted from an ineffective drug dosage (INR <2), while DRP6 was caused by an unsafe drug dosage (INR >3.5). DRP5 (non-quantitative safety of drugs) was the most prevalent, accounting for 62.32% (172) of all identified DRPs. DRP5 included 27 adverse drug reactions and 145 drug interactions, respectively. The ADRs were all bleeding-related events, such as ecchymosis, gastrointestinal haemorrhage, blood urine, and haemoptysis.

### 3.2 Risk of drug combined with warfarin on haemorrhage

Additionally, we analyzed the U.S. FAERS dataset to select newly approved drugs that interact with warfarin and affect its safety, focusing on those that increase or decrease the risk of bleeding (Table 3). A total of 53,709 reports of adverse reactions from patients using warfarin were collected, with most originating from the United States. More than half of these reports (n = 29,017) were related to bleeding events, with an average age of 71 ± 15 years. The outcomes of the adverse reactions were severe, with 56.22% of the reports resulting in “Hospitalization - initial or prolonged”.

**TABLE 3:** Characteristics of cases between haemorrhages and non-haemorrhages group (N = 53,709).

Characteristic	Non-haemorrhages (n = 24,692)	Haemorrhages (n = 29,017)
Age	67 ± 17	71 ± 15
missing	10,071	6,755
Sex		
Male	9,115 (42.4%)	14,348 (55.5%)
Female	12,359 (57.6%)	11,514 (44.5%)
missing	3,218	3,155
Outcome		
Death/Life-threatening	3,217 (20.4%)	5,139 (20.5%)
Hospitalization – initial or prolonged	6,375 (40.5%)	14,068 (56.2%)
Disability	168 (1.1%)	122 (0.5%)
Congenital anomaly	61 (0.4%)	5 (0.0%)
Required Intervention	73 (0.5%)	251 (1.0%)
Others	5,866 (37.2%)	5,435 (21.7%)
missing	8,932	3,997
Reporter occupation		
Drug consumer (CN)	12,039 (48.8%)	8,365 (28.8%)
Pharmacist (PH)	1,962 (7.9%)	8,479 (29.2%)
Other health-professional (OT)	4,181 (16.9%)	4,988 (17.2%)
Physician (MD)	4,160 (16.8%)	4,665 (16.1%)
Lawyer (LW)	29 (0.1%)	16 (0.1%)
Registered Nurse (RN)	6 (0.0%)	8 (0.0%)
unknown	588 (2.4%)	958 (3.3%)
Country (top six)		
United States	13,704 (55.5%)	10,255 (35.3%)
Italy	1,008 (4.1%)	2,355 (8.1%)
United Kingdom	934 (3.8%)	1,845 (6.4%)
France	752 (3.0%)	1,465 (5.0%)
Canada	912 (3.7%)	430 (1.5%)
Japan	411 (1.7%)	705 (2.4%)
unknown	5,466 (22.1%)	10,298 (35.5%)

Values are n, n (%), or Mean ± SD.

**FIGURE 2:**
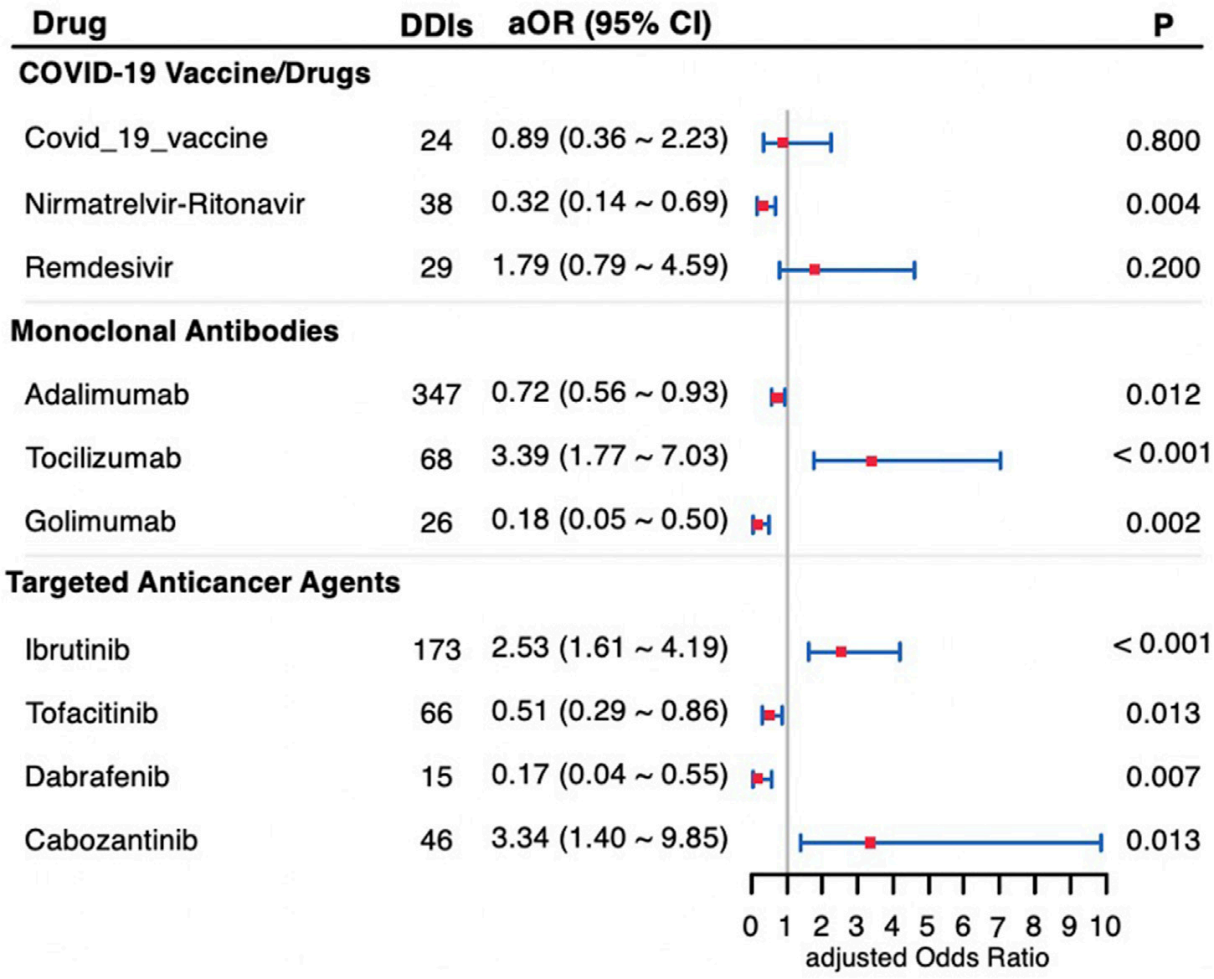
Forest plot of the bleeding risk of drug-drug interactions with warfarin adjusted for sex and age. DDIs, drug-drug interactions; aOR, adjusted Odds Ratio; 95% CI, 95% confidence interval.

The coadministration of drugs that interact with warfarin can significantly affect its efficacy and elevate the risk of adverse reactions. The most common interactions involved potentiation or inhibition of warfarin, which can lead to an elevated or reduced risk of bleeding. We analyzed the effects on bleeding risk of concomitant use of warfarin with 10 recently approved medications, including COVID-19 vaccines/drugs, monoclonal antibodies, and targeted anticancer agents. Table 4 (crude model) and Figure 2 (adjusted model) shows the key findings. Significantly, coadministration with tocilizumab (aOR: 3.39 [1.77–7.03]; *P* < 0.001), ibrutinib (aOR: 2.53 [1.61–4.19]; *P* < 0.001), or cabozantinib (aOR: 3.34 [1.40–9.85]; *P* = 0.013) increased the risk of bleeding. In contrast, the concurrent use of warfarin with nirmatrelvir-ritonavir (aOR: 0.32 [0.14–0.69]; *P* = 0.004), adalimumab (aOR: 0.72 [0.56–0.93]; *P* = 0.012), golimumab (aOR: 0.18 [0.05–0.50]; *P* = 0.002), tofacitinib (aOR: 0.51 [0.29–0.86]; *p* = 0.013), or dabrafenib (aOR: 0.17 [0.04–0.55]; *P* = 0.007) showed a statistically significant reduction in bleeding risk. Remdesivir exhibited a significant increase in bleeding risk in the crude model (*P* = 0.047, chi-square test); No significant effect on bleeding risk was observed with remdesivir after adjustment (aOR: 1.79; *P* = 0.2) or the COVID-19 vaccine (aOR: 0.89; *P* = 0.8).

**TABLE 4:** Bleeding risk of drug interactions with warfarin for ten medications.

Drug	DDIs, n	P	Crude OR	95% CI
COVID-19 Vaccine/Drugs				
Covid_19_vaccine	24	0.700	0.85	0.38, 1.91
Nirmatrelvir-Ritonavir	38	0.005	0.39	0.19, 0.76
Remdesivir	29	0.047	2.23	1.03, 5.37
Monoclonal Antibodies				
Adalimumab	347	0.011	0.76	0.62, 0.94
Tocilizumab	68	<0.001	2.77	1.62, 5.01
Golimumab	26	<0.001	0.15	0.05, 0.40
Targeted Anticancer Drugs				
Ibrutinib	173	<0.001	2.84	2.01, 4.09
Tofacitinib	66	0.001	0.43	0.25, 0.70
Dabrafenib	15	0.008	0.21	0.05, 0.67
Cabozantinib	46	0.007	2.41	1.29, 4.86

DDIs, drug-drug interactions; OR, odds ratio; 95% CI, 95% confidence interval.

## 4 Discussion

Warfarin is an indispensable component of oral anticoagulant therapy for certain anticoagulant treatments and prevention, such as atrial fibrillation, post-mechanical valve replacement, and patients with severe renal insufficiency (Hindricks et al., 2021). The analysis of inpatient EMRs revealed that drug interactions with warfarin present the highest safety risk, with adverse reactions primarily resulting in bleeding events and severe outcomes. Therefore, identifying and alerting patients or healthcare providers to novel drug interaction signals is necessary to mitigate the frequent occurrence of interactions and ensure the safe clinical use of warfarin.

The clinical use of warfarin presented challenges due to inaccurate dosing, adverse reactions, and drug interactions. Among these, the interactions between warfarin and other drugs pose the most significant problem. Warfarin interacts with various drugs through multiple mechanisms that influence its anticoagulant effects. The primary mechanism involves hepatic metabolism via the cytochrome P450 (CYP) enzyme system. The S-enantiomer of warfarin is more effective and primarily metabolized by CYP2C9, while the less active R-enantiomer is metabolized by CYP3A4 and CYP1A2. CYP inhibitors, such as fluconazole, reduce warfarin metabolism and increase the risk of bleeding, while inducers, such as rifampin, accelerate its metabolism, decreasing its anticoagulant efficacy. Additionally, competition for plasma protein binding, particularly with drugs such as NSAIDs, increases free warfarin levels, thereby enhancing its effects. Furthermore, medications that interfere with vitamin K absorption, such as cephalosporins, potentiate the anticoagulant activity of warfarin. Overall, the clinical application of warfarin is complicated by individual variability and frequent DDIs. We analyzed ten new drugs approved for marketing in the past decade which have limited clinical experience postapproval. These medications are not listed on the warfarin drug labels. Some of them are reported to induce adverse reactions when combined with warfarin, raising suspicions of drug interactions, but no confirmations or consistent conclusions exist.

COVID-19 vaccine/drugs are newly introduced treatments available for the past 4 years, in response to the SARS-CoV-2 pandemic. These medications have limited clinical trial data and relatively insufficient reports. A study on outpatients who were administered the COVID-19 vaccine BNT162b2 reported an increased risk of bleeding (indicated by INR ≥5) when taking warfarin (Visser et al., 2022). Here, the outcome included bleeding tendencies indicated by laboratory tests and bleeding in organ tissues, which provide a more scientific measure than INR alone. Mungmunpuntipantip and Wiwanitkit (2022) noted that the COVID-19 vaccine increases immunoglobulin levels in the blood, which could raise blood viscosity and potentially influence laboratory coagulation results. Another case-crossover study, which only included only adolescents and young adults, showed INR fluctuations with no difference observed in complications after vaccination (Visser et al., 2023). This aligns with the conclusion of our study. Nirmatrelvir-ritonavir, launched in December 2021, contains ritonavir, a potent inhibitor of cytochrome P450 3A4 (CYP3A4) and P-glycoprotein. Coadministation of ritonavir with nirmatrelvir increases the blood concentration of nirmatrelvir. Additionally, conflicting case series regarding interactions between warfarin and nirmatrelvir-ritonavir exist (Abraham et al., 2022; Muse et al., 2022). Our results indicated that the coadministration of warfarin and nirmatrelvir-ritonavir reduces the risk of bleeding (aOR = 0.32, Figure 2). In contrast, Remdesivir, another anti-CoV-19 virus drug launched in May 2020, increases warfarin bleeding events (*P* = 0.047, Table 4). To data, only case reports showed that co-prescription of the drugs causes abnormally elevated INR in patients (Manigaba et al., 2020; Yao et al., 2021; Landayan et al., 2022).

Monoclonal Antibodies are a type of biological preparation used for the targeted treatment of rheumatoid arthritis (RA). Among them, adalimumab and golimumab are TNF-α inhibitors, while tocilizumab is an IL-6 inhibitor. TNF-α, IL-1, and IL-6 blockers could theoretically reduce the effect of warfarin by inducing cytochrome P450 enzymes (Cheemalavagu et al., 2020; Khiali and Entezari-Maleki, 2020), but empirical data on this interaction reamins scarce. In our study, adalimumab and golimumab align with this inference, while tocilizumab shows an increased risk of bleeding with warfarin (aOR = 3.39, Figure 2). Recently, a case report of a patient who taking tocilizumab and warfarin simultaneously developed a spontaneous spinal epidural hematoma (Gunes et al., 2020), and the evidence was corroborated by our study which suggest that their combined use may increase the bleeding risk.The effect of tocilizumab on coagulation parameters and CYP enzymes warrants further experimental study.

Targeted anticancer drugs are enzyme inhibitors that block one or more protein kinases representing a major class of new anticancer drugs in clinical practice. A retrospective cohort study showed that the risk of bleeding increased when ibrutinib was used with warfarin (Allouchery et al., 2023), consistent with the findings of our study. A clinical study involving 60 patients found that dabrafenib reduced INR when used with warfarin but did not address the bleeding risk (Suttle et al., 2015). Similarly, tofacitinib was related to pulmonary embolism in a patient with ulcerative colitis (Anuj and Patel, 2020), but no conmbination result was observed. While cabozantinib was reported to cause bleeding when used with apixaban (Santini et al., 2019) and had a case report of INR elevation with warfarin (Foxx-Lupo et al., 2016), the relationship between these drugs and warfarin is still under study. This study provides a data reference for the medication risk of tumor patients taking ibrutinib, tofacitinib, dabrafenib, and cabozantinib in combination with warfarin anticoagulation. The interactions observed with these medications illustrate the complexity of the pharmacokinetics and pharmacodynamics associated with warfarin, highlighting the critical need for postmarket monitoring and validation to ensure optimal anticoagulation and minimize adverse events when co-administered with new medications. These findings serve as early warnings that assist in risk assessment for comedication. Meanwhile, the limited availability of existing references underscores the need for further investigation to clarify the underlying mechanisms based on these results.

Nevertheless, this study had some limitations. The study only examined the pairwise interactions between warfarin and other drugs. However, many patients, particularly the elderly, take multiple drugs, making the interactions among multiple medications a more complex issue. Additionally, the study analyses suspected interactions identified by medical professionals and pharmaceutical companies to gather more accurate information. This approach may overlook other potential drug interactions. Deep learning methods are well-suited for extracting extensive information from big data, enabling a comprehensive investigation. Furthermore, retrospective data sources, such as EMR and the FAERS have limitations. Given their short market presence, COVID-19 drugs may not have fully shown all potential interactions with warfarin. Therefore, rigorous causal inference requires validation through prospective studies. Further research on these interactions is anticipated.

In conclusion, the EMR results showed that the main issues with warfarin are linked to DRP5, specifically drug interactions. Additionally, we examined big data from FAERS to assess the relationship between newly marketed drugs and their combined medication with warfarin regarding the risk of bleeding. Our findings indicated that the combination of tocilizumab, ibrutinib, and cabozantinib with warfarin significantly increases the risk of bleeding. Conversely, nirmatrelvir-ritonavir, adalimumab, golimumab, tofacitinib, and dabrafenib significantly reduce the risk of bleeding when combined with warfarin. Additionally, this study presented that the bleeding ADRs of remdesivir are significantly higher than its non-bleeding ADRs, although no difference was observed after adjusting for gender and age. Finally, our study showed no significant effect of the COVID-19 vaccine on bleeding risk. This study investigated the relationship between ten medications and their interactions with warfarin, which could lead to bleeding, providing real-world evidence for the safety of using these medications in combination with warfarin.

## Data Availability

The original contributions presented in the study are included in the article/supplementary material, further inquiries can be directed to the corresponding author.
